# 

**Published:** 1971-07

**Authors:** 


### CONTENTS

| | |
|---|---|
| **Beethoven (Muss es sein? Es muss sein!)** ... <br> Beryl D. Corner | 43 |
| County Maps of the West Country ... ... <br> J. P. Mitchell | 51 |
| The Population Explosion—a personal Opinion <br> J. C. Briggs | 59 |
| Obituary ... ... ... ... ... ... | 61 |
| Society Report ... ... ... ... ... | 62 |
| Wisdom from the Past ... ... ... ... | 63 |
| The Medical Library ... ... ... ... | 64 |
| News and Notes ... ... ... ... ... | 66 |

F. Bailey & Son Ltd., Dursley, Glos.

